# The LuxS/AI-2 Quorum-Sensing System of *Streptococcus pneumoniae* Is Required to Cause Disease, and to Regulate Virulence- and Metabolism-Related Genes in a Rat Model of Middle Ear Infection

**DOI:** 10.3389/fcimb.2018.00138

**Published:** 2018-05-04

**Authors:** Mukesh K. Yadav, Jorge E. Vidal, Yoon Y. Go, Shin H. Kim, Sung-Won Chae, Jae-Jun Song

**Affiliations:** ^1^Department of Otorhinolaryngology-Head and Neck Surgery, Korea University College of Medicine, Seoul, South Korea; ^2^Institute for Medical Device Clinical Trials, Korea University College of Medicine, Seoul, South Korea; ^3^Hubert Department of Global Health, Rollins School of Public Health, Emory University, Atlanta, GA, United States

**Keywords:** *Streptococcus pneumoniae*, LuxS/AI-2, quorum-sensing, *luxS* mutation, biofilm, *in vivo* colonization

## Abstract

**Objective:** *Streptococcus pneumoniae* colonizes the nasopharynx of children, and from nasopharynx it could migrate to the middle ear and causes acute otitis media (AOM). During colonization and AOM, the pneumococcus forms biofilms. *In vitro* biofilm formation requires a functional LuxS/AI-2 quorum-sensing system. We investigated the role of LuxS/AI-2 signaling in pneumococcal middle ear infection, and identified the genes that are regulated by LuxS/AI-2 during pneumococcal biofilm formation.

**Methods:** *Streptococcus pneumoniae* D39 wild-type and an isogenic D39Δ*luxS* strain were utilized to evaluate *in vitro* biofilm formation, and *in vivo* colonization and epithelial damage using a microtiter plate assay and a rat model of pneumococcal middle ear infection, respectively. Biofilm structures and colonization and epithelial damage were evaluated at the ultrastructural level by scanning electron microscopy and confocal microscopy. Microarrays were used to investigate the global genes that were regulated by LuxS/AI-2 during biofilm formation.

**Results:** The biofilm biomass and density of D39Δ*luxS* were significantly (*p* < 0.05) lower than those of D39 wild-type. SEM and confocal microscopy revealed that D39Δ*luxS* formed thin biofilms *in vitro* compared with D39 wild-type. The *in vivo* model of middle ear infection showed that D39Δ*luxS* resulted in ~60% less (*p* < 0.05) bacterial colonization than the wild-type. SEM analysis of the rat middle ears revealed dense biofilm-like cell debris deposited on the cilia in wild-type D39-infected rats. However, little cell debris was deposited in the middle ears of the D39Δ*luxS*-inoculated rats, and the cilia were visible. cDNA-microarray analysis revealed 117 differentially expressed genes in D39Δ*luxS* compared with D39 wild-type. Among the 66 genes encoding putative proteins and previously characterized proteins, 60 were significantly downregulated, whereas 6 were upregulated. Functional annotation revealed that genes involved in DNA replication and repair, ATP synthesis, capsule biosynthesis, cell division, the cell cycle, signal transduction, transcription regulation, competence, virulence, and carbohydrate metabolism were downregulated in the absence of LuxS/AI-2.

**Conclusion:** The *S. pneumoniae* LuxS/AI-2 quorum-sensing system is necessary for biofilm formation and the colonization of the ear epithelium, and caused middle ear infection in the rat model. LuxS/AI-2 regulates the expression of the genes involved in virulence and bacterial fitness during pneumococcal biofilm formation.

## Introduction

Otitis media (OM) is one of the main reasons antibiotics are prescribed for children in both developing and developed countries (Grijalva et al., 2009; Arguedas et al., 2010). More than 80% of children up to the age of 3 year experience at least one episode of acute OM (AOM), and the health and economic burdens associated with AOM are significant (Pichichero, 2013; Usonis et al., 2016). *Streptococcus pneumoniae* (*S. pneumoniae*) is the most important bacteria that causes AOM in children (Bergenfelz and Hakansson, 2017). Although *S. pneumoniae* can cause disease, it is a commensal bacterium that quiescently and asymptomatically colonizes the mucosal surface of the nasopharynx in the form of a specialized structure called a biofilm (Bogaert et al., 2004; Simell et al., 2012). Once established as a biofilm, the bacteria can disperse to other typically sterile anatomical sites and cause pneumonia, OM, bacteremia, or meningitis (Hall-Stoodley et al., 2006; Sanchez et al., 2010; Weimer et al., 2010; Ash and Sheffield, 2013; Pichichero, 2013; Shak et al., 2013). It has been suggested that pneumococcal biofilms can also asymptomatically colonize the mucosal surfaces of the middle ear (during OM) and sinuses (during rhinosinusitis) (Hall-Stoodley et al., 2006; Sanderson et al., 2006; Hoa et al., 2009). During colonization and biofilm formation, the pneumococci replicate slowly and express low levels of virulence factors, such as the polysaccharide capsule. They also produce extracellular DNA, proteins, lipids, and polysaccharides.

The bacteria within biofilms are embedded in a self-produced extracellular polymeric substance (EPS) matrix, and are resistant to both host immune defenses and antibiotics (Donlan and Costerton, 2002). More frequent genetic transformation has also been detected in biofilms. The available DNA in the biofilm matrix serves as a substrate for transformation that can result in the evolution of resistant strains and the spread of drug-resistant genotypes (Trappetti et al., 2011c; Vidal et al., 2011; Croucher et al., 2012; Chao et al., 2014). It has been reported that the changes observed in pneumococcal transcription during biofilm formation are also associated with colonization (Sanchez et al., 2011). Furthermore, bacteria dispersed in biofilms have an increased propensity for tissue dissemination and pathogenesis compared with bacteria in free-floating, planktonic culture (Marks et al., 2013; Chao et al., 2014). The biofilm mode of growth provides an opportunity for pneumococci to colonize the upper respiratory tract and persist without causing disease; thus, pneumococcal carriage is common (Simell et al., 2012; Shak et al., 2013; Gilley and Orihuela, 2014). The persistence, pathogenesis, and drug resistance of pneumococcal biofilms is of high clinical importance.

During biofilm formation, bacteria regulate gene expression in response to changes in population density through a mechanism called quorum sensing (QS) (Hense et al., 2007). QS is mediated by secreted molecules called auto-inducers (AIs). One of these, a furanosyl borate diester called AI-2, is a metabolic byproduct of a *luxS* gene-encoded synthase: an enzyme involved primarily in the conversion of ribosyl-homocysteine into homocysteine and 4,5-dihydroxy-2,3-pentanedione (DPD), which is the precursor of AI-2 (Chen et al., 2002; Trappetti et al., 2011a). Various studies have shown that LuxS regulates pneumococcal biofilm formation, competence, and autolysis (Trappetti et al., 2011c; Vidal et al., 2011).

Previous studies using a mouse model of pneumococcal colonization suggest that LuxS plays an important role in persistence in the nasopharynx (Joyce et al., 2004). It has also been reported that *S. pneumoniae luxS* mutant strain has low *in vitro* biofilm formation capacity, and is defective in genetic competence and iron uptake (Trappetti et al., 2011c; Vidal et al., 2011, 2013). Using a mouse model of intranasal challenge, Stroeher et al. (2003) demonstrated that the ability to spread from the nasopharynx to the lungs or blood was reduced in an *S. pneumoniae* D39 *luxS* mutant compared with the wild-type D39 strain (Stroeher et al., 2003). More recently, it has been reported that LuxS/AI-2 signaling enables pneumococci to use galactose as a carbon source, and enhances capsular polysaccharide production and the hyper-virulent phenotype (Trappetti et al., 2017). However, the role of the LuxS/AI-2 QS system in the global gene expression of pneumococcal biofilms, and in the *in vivo* colonization of the rat middle ear has not been reported. Therefore, in the present study we confirmed that LuxS/AI-2 is necessary for *in vitro* biofilm formation, analyzed its ultrastructure by electron microscopy, and assessed the effects of the absence of LuxS/AI-2 signaling on pneumococcal middle ear infection. We used a rat model of pneumococcal middle ear infection for the *in vivo* studies, and ultimately identified the global genes that are regulated by LuxS/AI-2 during pneumococcal biofilm formation.

## Materials and methods

### Ethics statement

The animal experiment protocol was approved by the Institute Review Board of Korea University, Guro Hospital, Seoul, South Korea. The animal experiments were carried out as per the guidelines provided by the Animal Research Committee, Korea University College of Medicine, Seoul, South Korea.

### Bacterial strains and culture media

*Streptococcus pneumoniae* D39 (NCTC 7466) was obtained from the Health Protection Agency Culture Collection (Salisbury, UK). It is Avery's Virulent Serotype 2 encapsulated strain, and is extremely virulent in animal models of infection (Avery et al., 1944). The *S. pneumoniae luxS* mutant strain (D39Δ*luxS*) has been prepared and characterized in previous studies (Vidal et al., 2011, 2013). The *S. pneumoniae* strains were grown on blood agar plates (BAPs) (Shin Yang Chemicals Co., Ltd., Seoul, Korea) and in brain heart infusion (BHI) broth.

### *In vitro* biofilm formation abilities of *S. pneumoniae* D39 wild-type and D39Δ*luxS*

The *in vitro* biofilm formation abilities of *S. pneumoniae* D39 wild-type and an isogenic D39Δ*luxS* strain were evaluated using a static microtiter plate assay, as described previously (Christensen et al., 1982; Yadav et al., 2017b). Briefly, the pneumococcal strains were grown on BAPs overnight. A single colony from each plate was transferred to BHI broth and grown to the mid-exponential phase. The log-phase cells were diluted (1:200), and 1 mL of each cell suspension was seeded into a 24-well polystyrene flat-bottomed microtiter plate (BD Falcon, Sparks, MD, USA), and incubated at 37°C for various times. After incubation, the planktonic cells and medium were removed, and the biofilms remaining in the wells were washed twice with phosphate-buffered saline (PBS). The biofilms were then stained with 200 μL of 0.1% crystal violet (CV) for 15 min. After staining, the plates were washed twice with PBS and air-dried. The CV in the wells was dissolved in 1 mL of ethanol, 200 μL of the CV solution from each well was transferred to a 96-well plate, and the absorbance at 570 nm was measured using a micro-plate reader.

Alternative, the viable bacterial within the biofilms were detected by cfu counting. The biofilms were washed twice with PBS and suspended by sonication in 1 mL of PBS. The resulting biofilm suspensions were serially diluted and plated onto BAPs, and the bacteria were counted after overnight incubation at 37°C.

### Effect of incubation time on the *in vitro* biofilm growth of *S. pneumoniae* D39 wild-type and D39Δ*luxS*

It has been reported that *S. pneumoniae* biofilms are affected by incubation time. Therefore, to evaluate the effect of incubation time on *in vitro* biofilm formation, we grew *S. pneumoniae* D39 wild-type and D39Δ*luxS* biofilms for various times (6, 12, 18, and 24 h). We then determined biofilm biomass using a CV-microtiter assay, as described above.

### Scanning electron microscopy (SEM) analysis of *in vitro* biofilms formed by the *S. pneumoniae* D39 wild type and D39Δ*luxS*

We investigated the morphologies of the *in vitro*-formed biofilms of the D39 wild-type and the D39Δ*luxS* strains using SEM. The biofilms were grown for 18 h, as described above. After incubation, the biofilms were washed with PBS, and fixed with 2% glutaraldehyde and 2.5% paraformaldehyde. The biofilms were then treated with 1% osmic acid for 2 h, and dehydrated with a graded series of ethanol (60–95%). Biofilm samples were washed thrice with t-butyl alcohol (Sigma, St. Louis, MO, USA), and preserved under freezing conditions. The biofilm samples were freeze-dried using ES-2030 equipment (Hitachi, Tokyo, Japan), and coated with platinum using an ion coater (IB-5; Eiko, Kanagawa, Japan). SEM images were captured by field emission-SEM (FE-SEM, S-4700; Hitachi, Tokyo, Japan).

### Confocal microscopy of *S. pneumoniae* D39 wild-type and D39Δ*luxS* biofilms

*Streptococcus pneumoniae* D39 wild-type and D39Δ*luxS* biofilms were evaluated by confocal microscopy. The biofilms were grown on μ-slides (ibidi, Germany) for 18 h using the procedure described above. After incubation, the biofilms were stained using a LIVE/DEAD biofilm viability kit (Invitrogen, Carlsbad, CA, USA) according to the manufacturer's instructions. After washing with PBS, the stained biofilms were examined using a Nikon A1 confocal microscope (Nikon Instruments, Inc., NY, USA) with fluorescein (green) and Texas red (red) band-pass filter sets.

### Evaluation of the *in vivo* colonization capability of the *S. pneumoniae* D39 wild-type and D39Δ*luxS*

The *in vivo* colonization capability of the D39 wild-type and D39Δ*luxS* strains was evaluated using a rat model of OM (Yadav et al., 2012b, 2017a). Twenty healthy, pathogen-free Sprague Dawley (SD) rats weighing approximately 150–200 g were purchased from Koatech (Pyeongtaek, South Korea). All rats were housed isolated under sterile conditions for 2 weeks prior to the start of the experiments, and were examined for abnormalities in the middle ear. They were then divided into four groups: the rats in group 1 (*n* = 7) were inoculated with *S. pneumoniae* D39 wild-type; the rats in group 2 (*n* = 7) were inoculated with *S. pneumoniae* D39Δ*luxS*; the rats in group 3 (*n* = 3) received the medium only (vehicle control); and the rats in group 4 (*n* = 3) received no treatment (no procedure control). The rats were anesthetized with a combination of Zoletil H (tiletamine-zolazepam; Virbac, Carros, France) and Rompun H (xylazine-hydrochloride; Bayer, Leverkusen, Germany) at a ratio of 1:1. The bacteria suspensions containing the *S. pneumoniae* wild-type or Δ*luxS* strain were prepared in BHI medium, and 50 μL (~1 × 10^7^ colony-forming units (CFUs)) of the suspension (or medium only) was injected into the right middle ear of each rat in groups 1 (wild-type), 2 (Δ*luxS*), and 3 (medium) through the tympanic membrane (trans-tympanic membrane inoculation) using a tuberculin syringe and a 27-gauge needle. The rats were monitored daily for 1 week for abnormalities. The rats were then sacrificed, and bullae were acquired aseptically. The tympanic membranes and upper tissues were removed, and the middle ears were dissected and photographed. For the SEM analysis, representative bullae from each group were cleaned by trimming the bony parts so that the middle ear was clearly visible, and were preserved in SEM solution (glutaraldehyde and paraformaldehyde). To determine the number of viable bacteria, bullae from each group were aseptically homogenized with a mortar and pestle, serially diluted in PBS, and plated on BAP. After incubation at 37°C for 24 h, *S. pneumoniae* colonies were counted, and CFUs were calculated.

### Differential gene expression analysis of *in vitro* biofilms of *S. pneumoniae* D39 wild-type or D39Δ*luxS*

The global gene expression of *S. pneumoniae* D39 wild-type and D39Δ*luxS* biofilms were determined using a cDNA-microarray. For the experiment, *S. pneumoniae* D39 wild-type and D39Δ*luxS* biofilms were grown in 24-well plates for 18 h, as described above. The biofilms were washed twice with PBS, scraped, and suspended in PBS. The biofilm cells were then pelleted by centrifugation and treated with 100 μL of lysozyme [3 mg/mL in Tris-ethylenediaminetetraacetic acid (EDTA) buffer (TE); Sigma-Aldrich, St. Louis, MO, USA] for 4 min to lyse the cells. Total RNA was extracted using an RNeasy Total RNA Isolation System Kit (Qiagen, Valencia, CA, USA) according to the manufacturer's instructions. Contaminating DNA was removed by on-column RNase-free DNase (Qiagen) treatment for 10 min at 20–25°C. The quantity and quality of total RNA was detected using a NanoDrop (NanoDrop Technologies, Inc., Wilmington, DE, USA), and the integrity of the RNA was assessed using Bioanalyzer 2100 equipment (Agilent, Palo Alto, CA, USA).

RNA probe synthesis and hybridization were performed using the Agilent Low Input Quick Amp WT Labeling Kit according to the manufacturer's protocol. Briefly, 200 ng of total biofilm RNA was mixed with WT primer mix, and the samples were incubated at 65°C for 10 min. The cDNA master mix was then prepared with 5 × first strand buffer, 0.1 M dithiothreitol, 10 mM dNTP mix, and RNase Block Mix (AffinityScript), and added to the RNA + WT primer reaction mixture. The samples were incubated at 40°C for 2 h, and reverse transcription and dsDNA synthesis were terminated by incubation at 70°C for 15 min. The transcription master mix was prepared according to the manufacturer's protocol (5 × transcription buffer, 0.1 M dithiothreitol, NTP mix, T7-RNA polymerase blend, and cyanine 5-CTP in nuclease-free water). The transcription of dsDNA was performed by adding transcription master mix to the dsDNA reaction samples and incubating the mix at 40°C for 2 h. The amplified and labeled cRNA was purified on an RNase mini column (Qiagen) according to the manufacturer's protocol. The labeled complementary RNA (cRNA) target was quantified using an spectrophotometer.

After checking the labeling efficiency of the cyanine 5-labeled cRNA target, the cRNA was fragmented by adding 10 × blocking agent and 25 × fragmentation buffer, and incubating at 60°C for 30 min. The fragmented cRNA was resuspended in 2 × hybridization buffer and directly pipetted onto an assembled *S.pneumoniae*_6 x 7k V2 Microarray (mycroarray.com). The arrays were hybridized at 57°C for 17 h in an Agilent Hybridization oven. The hybridized microarrays were washed according to the manufacturer's washing protocol (Agilent Technology). After overnight incubation at 42°C, the slides were washed twice with washing solution 1 (containing 2 × saline-sodium citrate buffer (SSC) and 0.1% sodium dodecyl sulfate) for 5 min at 42°C, washed once with washing solution 2 (containing 0.1 × SSC and 0.1% sodium dodecyl sulfate) for 10 min at room temperature, and finally washed four times with 0.1 × SSC for 1 min at room temperature. The slides were dried by centrifugation at 650 rpm for 5 min. The hybridization image on the slide was scanned using 4000B apparatus (Axon Instruments, Union City, CA, USA).

The hybridization images were analyzed using GenePix Pro 3.0 software (Axon Instruments, Union City, CA, USA) to obtain the gene expression ratios of the D39 wild-type and D39Δ*luxS* biofilms. The microarray data were analyzed using Genowiz 4.0™ (Ocimum Biosolutions, Hyderabad, India), and normalized with Global LOWESS. The cutoffs for upregulated and downregulated genes were +2-fold and−2-fold, respectively. The microarray experiment was performed with three biological replicates. Statistical significance was calculated using Student's *t*-test, and *p*-values < 0.05 were considered significant. STRING version 10.5 (https://string-db.org) was used for functional annotation, and the UniProtKB database (http://www.uniprot.org/uniprot/P0A4M0) was used to search for clusters of biological processes in the gene ontology database within the two sets of differentially expressed genes (D39Δ*luxS* and D39 wild-type). The microarray data have been deposited in the National Center for Biotechnology Information (NCBI)'s Gene Expression Omnibus (GEO) database (http://www.ncbi.nlm.nih.gov/geo/info/linking.html), and are accessible through GEO Series accession number GSE109347.

### Real-time reverse transcription polymerase chain reaction (RT-PCR) analysis

To confirm the microarray data by real-time RT-PCR, we chose 15 genes that are differentially expressed in biofilms, and the 16S gene as a control. The primer sequences are presented in Table 1. Each 20-μL real-time RT-PCR reaction mixture included 10 μL of 2 × SYBR Green PCR Master Mix (Roche Applied Science, Indianapolis, IN, USA), 5 pmol each of the forward and reverse primers, and 2 μL of complementary DNA (cDNA). The PCR conditions were: an initial denaturation step at 95°C for 10 min, followed by 40 cycles of denaturation at 95°C for 15 s, and annealing and extension at 60°C for 1 min. Negative controls, which contained nuclease-free water instead of RNA, were included to confirm that the samples were free from contamination. To verify the absence of contaminating genomic DNA, each RT-PCR experiment included a no reverse transcriptase control. Relative gene expression was determined using the 2^−ΔΔCT^ method. The reference gene was 16S, and the standard condition was the D39 wild-type biofilm.

**Table 1:** List of primers used in the present study.

**Serial number**	**Gene**	**Primer sequences**	**Amplicon size (base pair)**
1	*16s*	5′-AACCAAGTAACTTTGAAAGAAGAC-′3	126
		5′-AAATTTAGAATCGTGGAATTTTT-′3	
2	*ply*	5′-TGAGACTAAGGTTACAGCTTACAG-′3	225
		5′-CTAATTTTGACAGAGAGATTACGA-′3	
3	*lytA*	5′-AGTTTAAGCATGATATTGAGAAC-′3	272
		5′-TTCGTTGAAATAGTACCACTTAT-′3	
4	*ccpA*	5′- GACAGGAAAAGGAATGAATGC-3′	116
		5′- GGAAACACCTGCTTCACGAG-3′	
5	*lacG-2*	5′-ACTAGCTGGTTCGGCAGTGT-3′	102
		5′-GCTTATCAAGCAGAAGGTGCT-3′	
6	*rnr*	5′-GCCTGATTTGACTCTTCGTG−3′	70
		5′-ACGGATACGGATCTGCTGAC−3′	
7	*argG*	5′-AAATCGCTTGGTTGGGATTA-3′	100
		5′-CACAAGCGTCAAGTCCTCAA-3′	
8	*comX2*	5′-GGCATGGTCTGCTTATTACATGA-3′	99
		5′-TCGATTTCGAAACTTGGTTTT-3′	
9	*mtnN*	5′-TTGCTGCTATGCCAGAAGAA-3′	76
		5′-TTCCCCAAAACAACTTGCTC-3′	
10	*cysK*	5′-ACTGGTGGAACGATTTCTGG-3′	119
		5′-TGAGGACCAGGTTTTTCACC-3′	
11	*ciaR*	5′-TGGATTTGATGTTGCCAGAA-3′	145
		5′-TAATCATCCGCTCCCAGTTC-3′	
12	*aliA*	5′-ATTGCCTTTGGTTTTGATCG-3′	173
		5′-TCCTTCCATTCATCCCCATA-3′	
13	*SufB*	5′-GCTAAGGGTGAGCCTGAGTG-3′	150
		5′-GGCTGGTTTGTCAGATGGTT-3′	
14	*blpU*	5′-GATTTTGCCAAAGCAGGTGT-3′	127
		5′-CATAGGCCACACCTCCAAGT-3′	
15	*glmU*	5′-GGACACAAGGCAGAATTGGT-3′	150
		5′-ATCTCCTGCAATGACCAAGG-3′	
16	*rafE*	5′-CGAAGGATGTCCATGACCTT-3′	161
		5′-GCAGATGCTTGGACACTCAA-3'	

## Results

### The *luxS* mutant strain formed less biofilm *in vitro*

**Figure 1:**
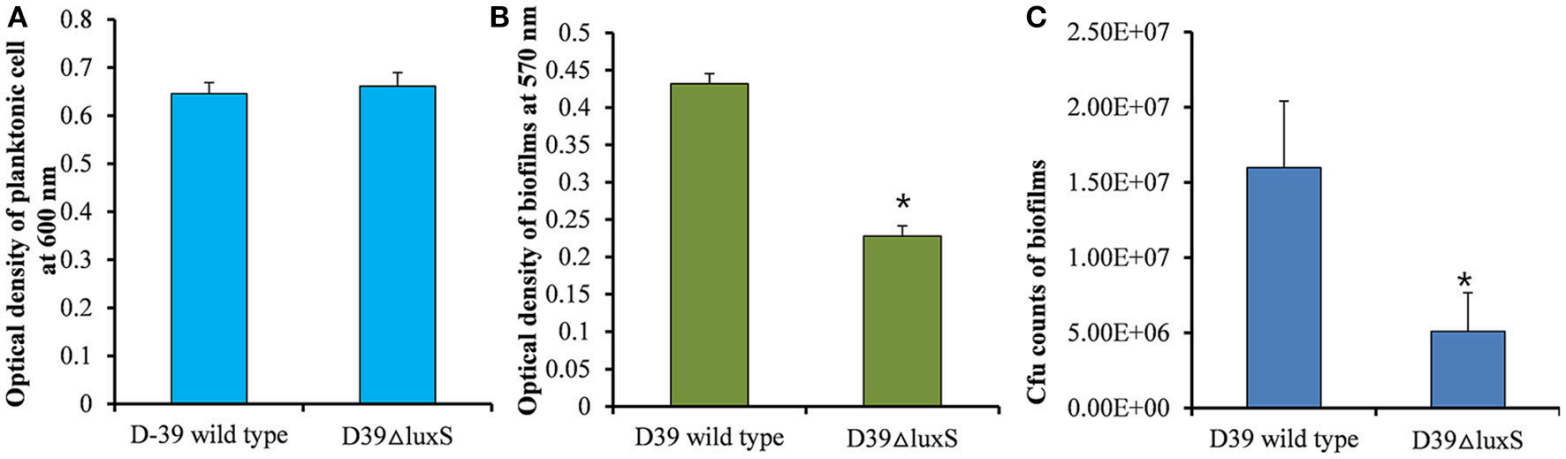
*Streptococcus pneumoniae* D39 wild-type and D39Δ*luxS* planktonic and biofilm growth. **(A)** Planktonic growth optical density at 600 nm. **(B)** Quantification of biomass of *in vitro* biofilms grown for 18 h, using a CV-microplate assay. **(C)** Colony-forming unit (CFU) counts of *in vitro* biofilms grown for 18 h. Error bars are the standard deviation from the mean. Statistical significance was calculated using the Student's *t*-test, ^*^p < 0.05.

It has been reported that the LuxS/AI-2 QS system regulates biofilm formation in *S. pneumoniae*. In the present study, we compared the planktonic growth and biofilm formation capability of *S. pneumoniae* D39 wild-type and the isogenic D39Δ*luxS* strain. No significant difference in the density of planktonic cells was observed between the D39 wild-type and D39Δ*luxS* strains (Figure 1A). After 18 h, the biofilm biomass of D39Δ*luxS* was significantly (*p* < 0.05) lower than that of the D39 wild-type when it was analyzed using the CV microplate assay (Figure 1B), or by bacterial counts (Figure 1C).

**Figure 2:**
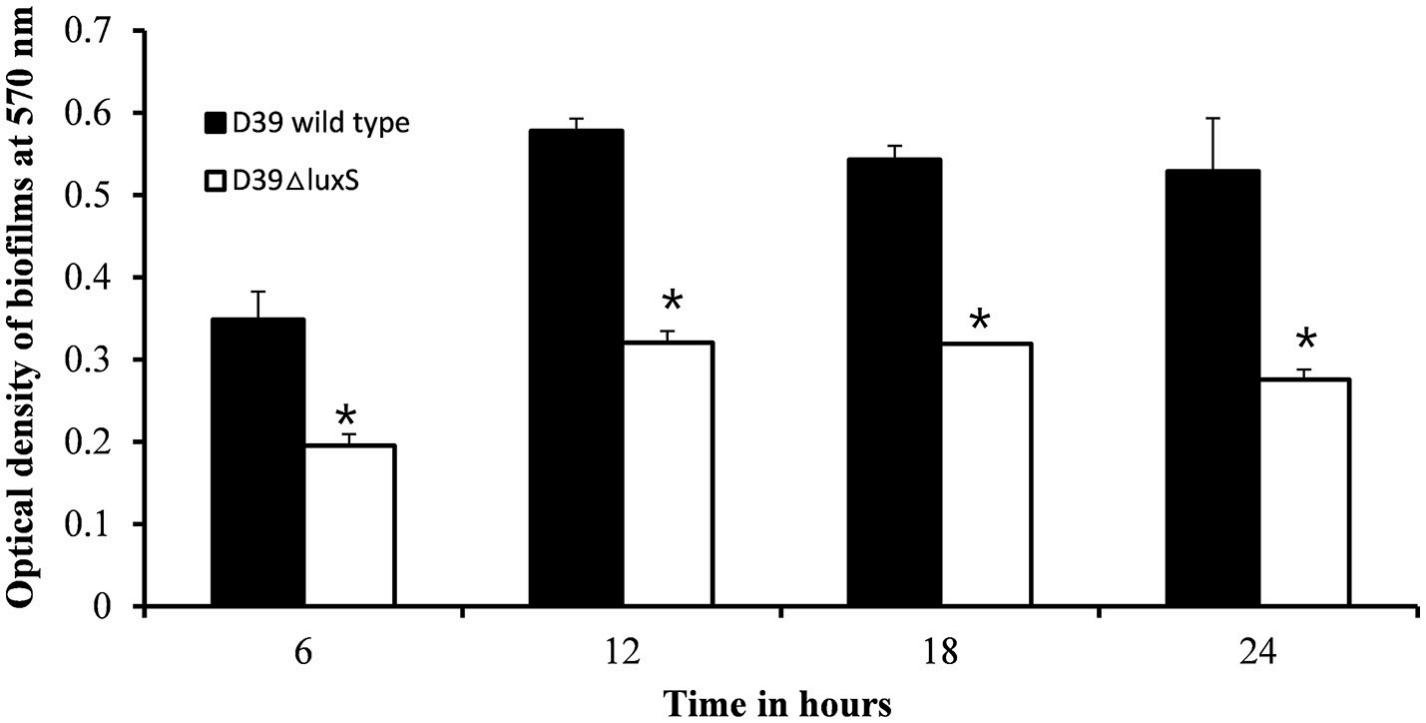
*In vitro* biofilm growth of *Streptococcus pneumoniae* D39 wild-type and D39Δ*luxS* strains at different time-points after inoculation (6, 12, 18, and 24 h). The error bars are the standard deviation from the mean. Statistical significance was calculated using the Student's *t*-test, ^*^p < 0.05.

We then conducted a time-course experiment to evaluate the effect of the absence of the *luxS* gene at various stages of biofilm formation. The results revealed a significant (*p* < 0.05) decrease in biofilm biomass in D39Δ*luxS* at 6, 12, 18, and 24 h post-inoculation (Figure 2). The results revealed that the D39Δ*luxS* mutant formed significantly less biofilm biomass than the D39 wild-type at all time-points.

### SEM revealed thin and scattered biofilms formed by the *luxS* mutant strain

**Figure 3:**
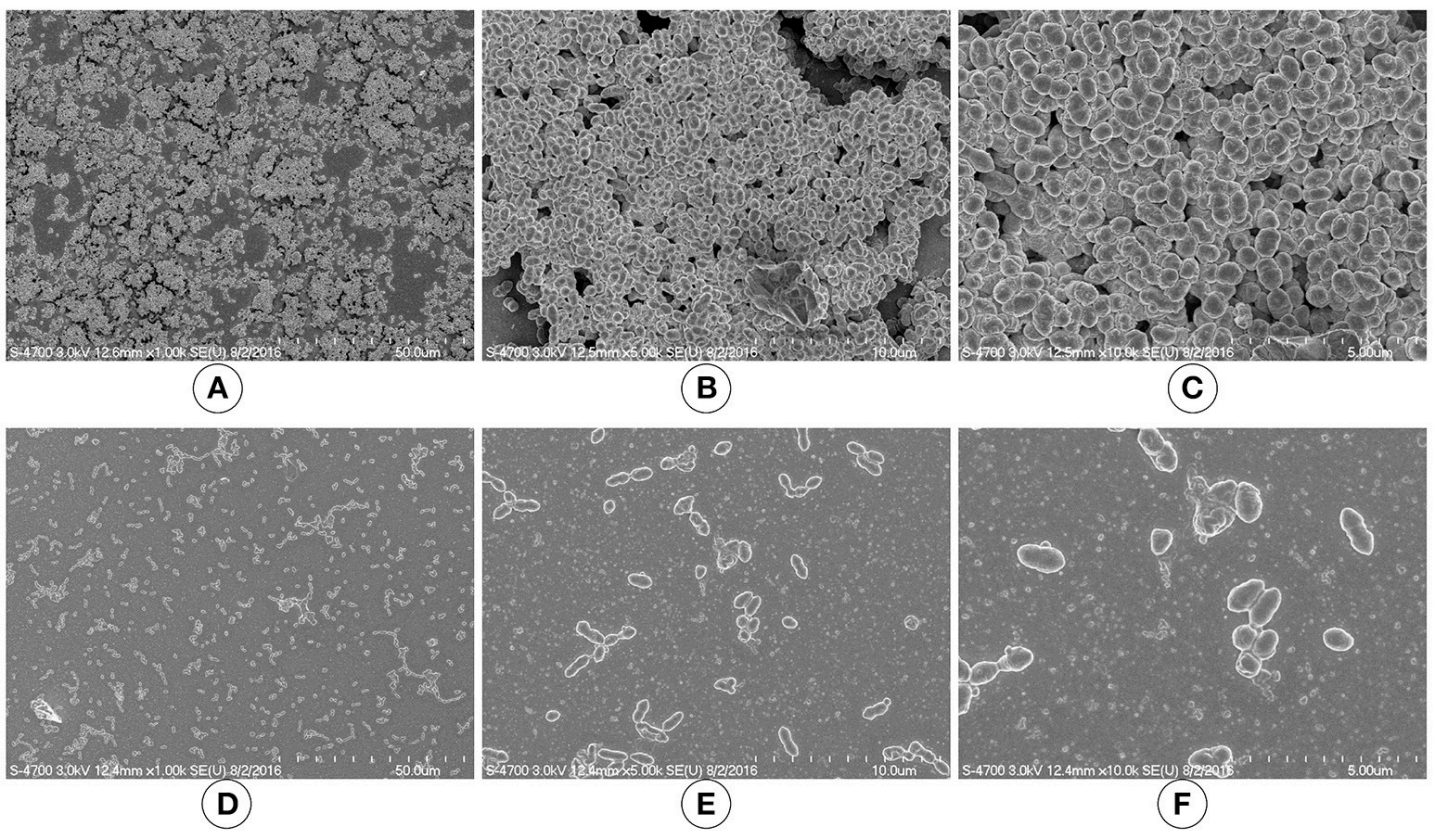
Scanning electron microscopy (SEM) images of *Streptococcus pneumoniae in vitro* biofilms grown for 18 h. **(A–C)** are representative SEM images of the D39 wild-type strain. The wild-type strain biofilms were thick and organized with a significant depth. **(D–F)** are SEM images of the D39Δ*luxS* strain. The D39Δ*luxS* biofilms were thin and disorganized, and extracellular polymeric substance (EPS) was absent.

We used SEM to investigate the morphologies of the D39 wild-type and D39Δ*luxS* biofilms grown for 18 h. The SEM analysis revealed that the D39 wild-type strain formed thick, three-dimensionally (3D) organized heterogeneous biofilms. The cells in the D39 wild-type biofilms were surrounded by extracellular polysaccharides (EPS), and were attached to both the bottom of the plate and to each other, forming an organized 3D biofilm structure with significant depth (Figures 3A–C). In contrast, the biofilms formed by D39Δ*luxS* were thin and disorganized. The cells were attached only to the base of the plate, and no cell–cell adherence was observed (Figures 3D–F). The cell surfaces were smooth and devoid of EPS.

### Confocal microscopy of *in vitro* biofilms formed by the D39Δ*luxS* mutant and D39 wild-type strains

**Figure 4:**
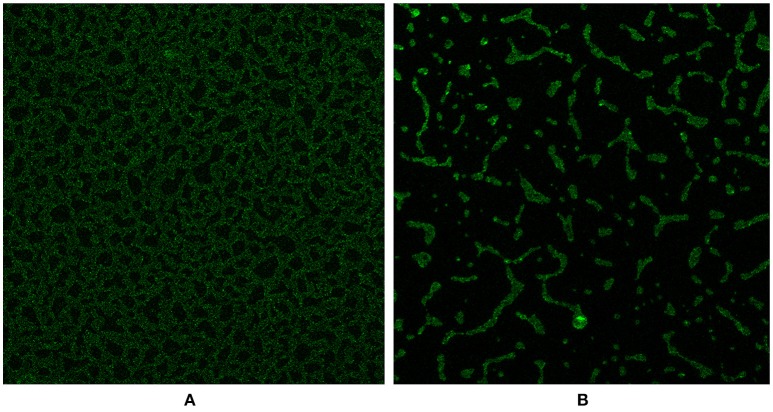
Confocal microscopy images of *Streptococcus pneumoniae in vitro* biofilms grown for 18 h. **(A)** Confocal microscopy image of the D39 wild-type strain biofilm. **(B)** Confocal microscopy image of the D39Δ*luxS* strain biofilm.

We investigated the D39Δ*luxS* and D39 wild-type biofilms grown for 18 h *in vitro* using a confocal microscope. Confocal microscopy revealed a significant difference in the morphology of the biofilms formed by the D39Δ*luxS* and D39 wild-type strains. The D39 wild-type biofilms were compact, thick, and had a well-organized 3D structure (Figure 4A). In contrast, the D39Δ*luxS* biofilms were thin with scattered pneumococci attached to the bottom of the dish. Their 3D structure was disorganized (Figure 4B).

### A mutation in *luxS* decreases colonization of *S. pneumoniae* in the rat middle ear

**Figure 5:**
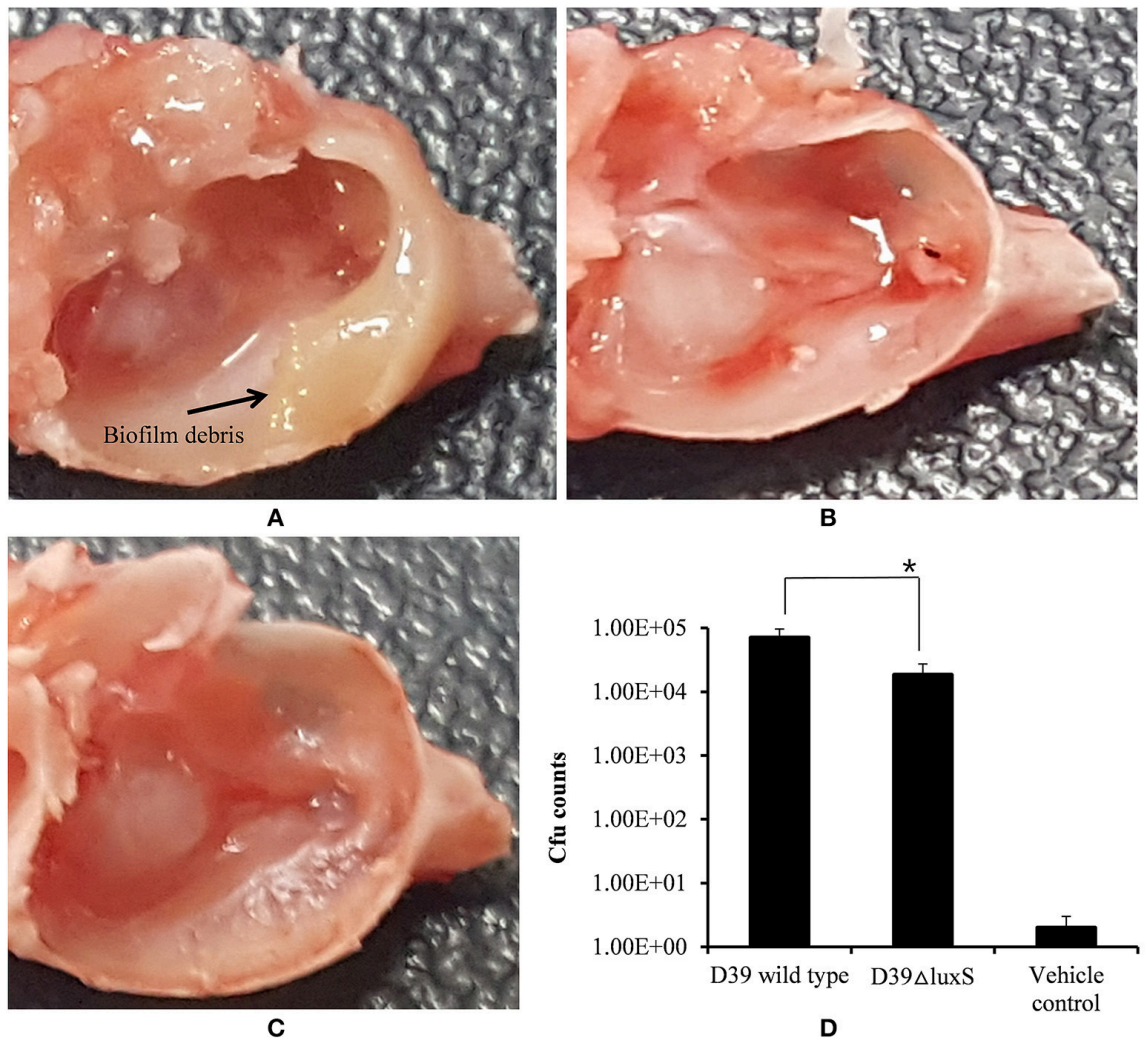
Digital photos of rat bullae and colony-forming unit (CFU) counts of bullae inoculated with *Streptococcus pneumoniae* D39 wild-type or D39Δ*luxS*. **(A)** Rat bullae inoculated with the D39 wild-type. **(B)** Rat bullae inoculated with the D39Δ*luxS*, and **(C)** Rat bullae inoculated with medium. **(D)** CFU of *S. pneumoniae* colonizing the rat middle ear mucosa. Error bars are the standard deviation from the mean. Statistical significance was calculated using the Student's *t*-test, ^*^p < 0.05.

The *in vivo* colonization abilities of the D39 wild-type and D39Δ*luxS* strains were evaluated using a rat model of middle ear infection. At 1 week post-inoculation, the rats were sacrificed, and their bullae were obtained, dissected, and cleaned of unwanted tissue. As Figure 5A shows, the rat bullae inoculated with the D39 wild-type were completely filled with biofilm-like debris, and exhibited severe mucosal swelling (Figure 5A). In contrast, the rat bullae inoculated with D39Δ*luxS* showed signs of inflammation, with a thick mucosa, but no visible biofilm-like debris (Figure 5B). As expected, the bullae of the control animals were clean, with no signs of inflammation (Figure 5C). The bacterial density in the middle ears of the rats inoculated with D39 wild-type was ~7.04 × 10^4^ (SD ± 26083.2) CFU/bullae, whereas that of the rats inoculated with the D39Δ*luxS* strain was significantly lower at ~1.85 × 10^4^ (SD ± 8859) CFU/bullae (Figure 5D). The mean CFUs of the D39Δ*luxS* strain were significantly lower (~60% reduction, *p* < 0.05) than the CFUs of the D39 wild-type strain.

**Figure 6:**
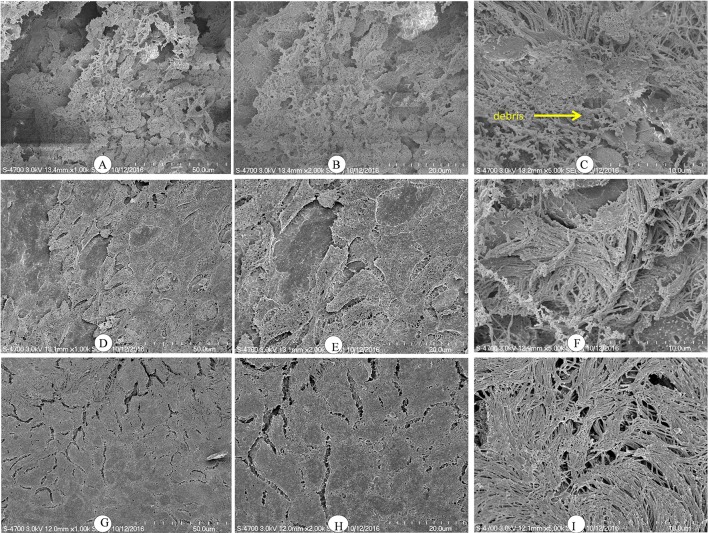
Scanning electron microscopy (SEM) images of rat bullae inoculated with *Streptococcus pneumoniae* D39 wild-type and D39Δ*luxS*. **(A–C)** are representative SEM images of rat bullae inoculated with the D39 wild-type strain. In rats colonized with the wild-type strain, dense biofilm/cell debris was deposited on the cilia, and the cilia were coagulated and completely covered with biofilm debris. **(D–F)** are representative SEM images of rat bullae inoculated with the D39Δ*luxS* strain. In rat bulla colonized with the D39Δ*luxS* strain, less biofilm debris was visible, although the cilia were coagulated. **(G–I)** are representative SEM images of rat bullae inoculated with medium (vehicle control). The vehicle control rat bulla were clean.

Histologically, the middle ear mucosa comprises both non-ciliated squamous epithelium and ciliated epithelium. The ciliated epithelium is distributed in the hypotympanum and Eustachian tube orifice, whereas the remainder of the middle ear bulla is covered with non-ciliated squamous epithelium. SEM images of a rat middle ear colonized by D39 wild-type are shown in Figures 6A–C. In these images, thick cells or biofilm debris deposits were visible (arrow). Cilia, however, were not visible but were completely covered with biofilm debris (Figures 6A–C). Conversely, in the rat middle ear colonized with D39Δ*luxS*, less biofilm debris was observed, although the cilia were coagulated (Figures 6D–F). The middle ears of the rats inoculated with vehicle (control) were clean, and neither biofilms nor cell debris were detected (Figures 6G–I).

### Differential gene expression in D39 wild-type biofilms and those produced by the D39Δ*luxS* mutant

**Figure 7:**
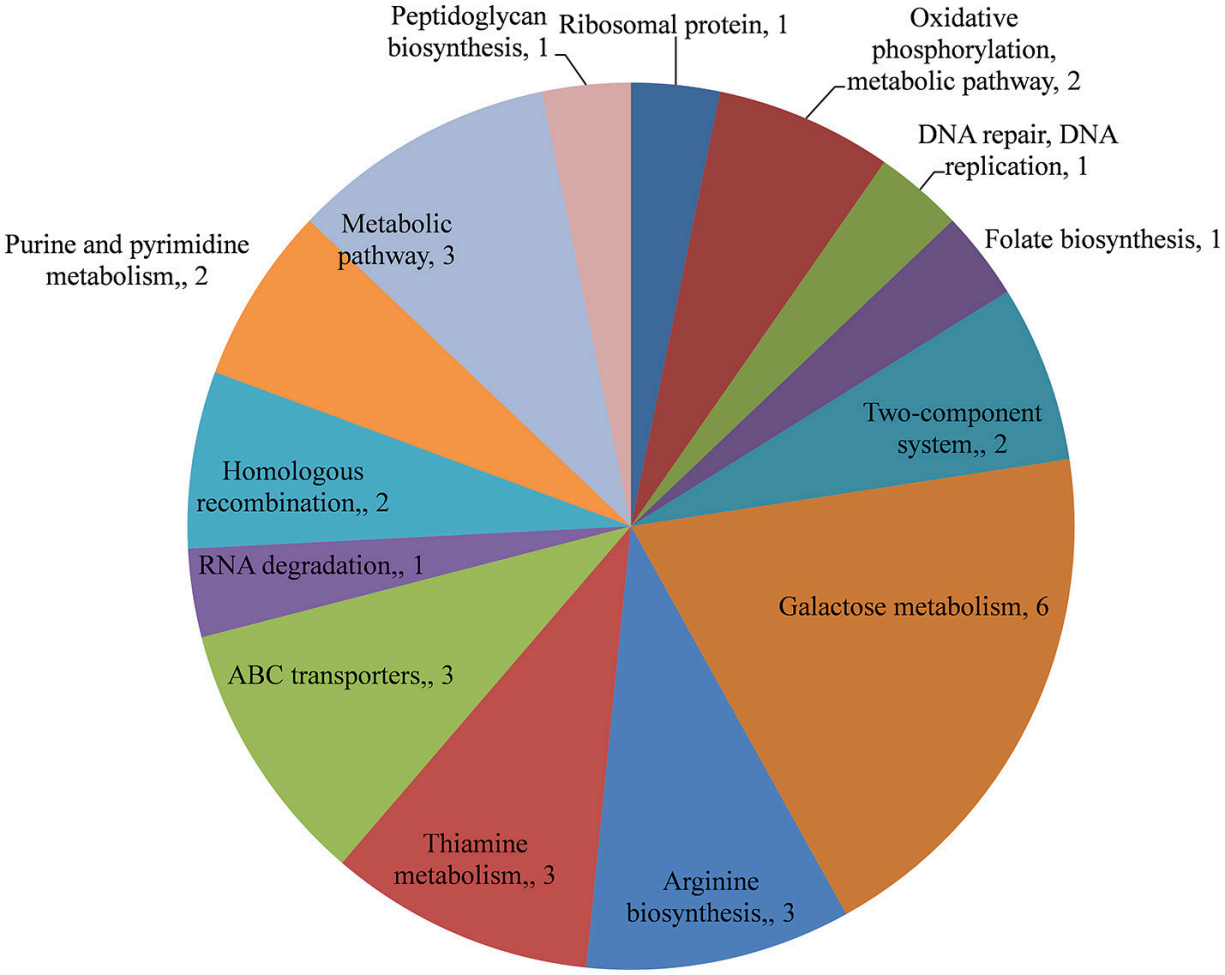
KEGG pathway analysis of genes downregulated in *Streptococcus pneumoniae* D39Δ*luxS* biofilms compared with the D39 wild-type biofilms.

The changes in the gene expression levels of the D39Δ*luxS* mutant biofilm with respect to D39 wild-type were evaluated using a cDNA microarray. Total RNA was extracted from 18-h-old biofilm samples grown in triplicate on a microtiter plate. The cDNA synthesis, labeling, and hybridization were performed as per standard protocols. The hybridization images were analyzed to detect the gene expression ratios in the D39 wild-type and D39Δ*luxS* biofilms. The fold changes in gene expression of the D39Δ*luxS* biofilms were detected with respect to the D39 wild-type. The ±2-fold changes in gene expression in the three biological replicate samples were included in this study. The cDNA-microarray analysis demonstrated that 117 genes were differentially expressed in D39Δ*luxS* compared with the D39 wild-type. According to functional group analysis, 51 of these genes were uncharacterized/hypothetical. Among the 66 genes encoding putative and previously characterized proteins, 60 were significantly downregulated and 6 were significantly upregulated (Table 2). The KEGG pathways of down-regulated genes in biofilms of *S. pneumoniae* D39Δ*luxS* with respect to D39 wild-type are shown in Figure 7.

**Table 2:** List of differentially expressed genes in biofilms of the *Streptococcus pneumoniae* D39*luxS* mutant strain compared with the wild-type D39.

**Serial number**	**Gene ID and gene name**	**Protein name**	**GO-biological process**	**Mean fold change in D39Δ*luxS***
1	SPD_0002 (*dnaN*)	DNA polymerase III subunit beta	DNA replication	−200
2	SPD_0013 (*ftsH*)	ATP-dependent zinc metalloprotease FtsH	Cell division	−800
3	SPD_0046 (*blpU*)	BacteriocinBlpU	Defense response to bacterium	−6.45
4	SPD_0065 (*bgaC*)	Beta-galactosidase 3	Carbohydrate metabolic process	−5.9
5	SPD_0071 (*galM*)	Aldose 1-epimerase	Hexose metabolic process	−2.2
6	SPD_0110 (*argG*)	Argininosuccinate synthase	Arginine biosynthetic process	−4.5
7	SPD_0195 (*rplW*)	50S ribosomal protein L23	Translation	−2.1
9	SPD_0261 (*pepC*)	Aminopeptidase C	Aminopeptidase activity	−4.76
10	SPD_0315 (cps2A)	Integral membrane regulatory protein Cps2A	DNA replication	−2.3
11	SPD_0316 (*cps2B*)	Tyrosine-protein phosphatase CpsB	Protein tyrosine phosphatase activity	−1.75
12	SPD_0317 (*cps2C*)	Chain length determinant protein/polysaccharide export protein, MPA1 family protein	Lipopolysaccharide biosynthetic process	−1.85
13	SPD_0318 (*cps2D*)	Tyrosine-protein kinase Cps2D cytosolic ATPase domain	Extracellular polysaccharide biosynthetic process	−2
14	SPD_0319 (*cps2E*)	integral component of membrane	Undecaprenylphosphateglucosephosphotransferase Cps2E	−1.8
15	SPD_0320 (*cps2T*)	Glycosyl transferase, group 1 family protein	Transferase activity	−2.4
16	SPD_0322 (*cps2G*)	Glycosyl transferase, group 1 family protein	Transferase activity, transferring glycosyl groups	−5.26
17	SPD_0334 (*aliA*)	Oligopeptide ABC transporter	ATP-binding cassette (ABC) transporter complex	−166
18	SPD_0468 (*blpR*)	Response regulator BlpR	Regulation of transcription	−166
19	SPD_0473 (*blpY*)	Immunity protein BlpY	Integral component of membrane	−2.7
20	SPD_0524 (*vncR*)	DNA-binding response regulator VncR	Regulation of transcription	−4.5
21	SPD_0532 (*recJ*)	Single-stranded-DNA-specific exonuclease RecJ	DNA repair	−200
22	SPD_0536 (*fibB*)	Beta-lactam resistance factor	Cell wall macromolecule biosynthetic process	−4.44
23	SPD_0578 (*pabB*)	Para-aminobenzoate synthase, component I	Folic acid-containing compound biosynthetic process	−4.5
24	SPD_0598 (*murD*)	UDP-N-acetylmuramoylalanine-d-glutamate ligase	Peptidoglycan biosynthetic process	4.52
25	SPD_0623 (*thiM*)	Hydroxyethylthiazole kinase 1	Thiamine biosynthetic process	−4.44
26	SPD_0654 (*livM*)	Branched-chain amino acid ABC transporter, permease protein	Transporter activity	−3.84
27	SPD_0701 (*ciaR*)	DNA-binding response regulator CiaR	Regulation of transcription	−200
28	SPD_0700 (*pepN*)	Aminopeptidase	Aminopeptidase activity	−4
29	SPD_0766 (*sufB*)	FeS assembly protein SufB	Iron-sulfur cluster assembly	−166
30	SPD_0813 (*nspC*)	Carboxynorspermidine decarboxylase	Nor-spermidine biosynthetic process	−144
31	SPD_0833 (*gid*)	Methylenetetrahydrofolate-tRNA-(uracil-5-)-methyltransferase TrmFO	tRNA processing	−200
32	SPD_0862 (*rnr*)	Ribonuclease R	Nucleic acid binding	−142
33	SPD_0866 (*pepF*)	Oligoendopeptidase F	Metalloendopeptidase activity	−5.2
34	SPD_0902 (*trmE*)	tRNA modification GTPaseMnmE	tRNA modification	−108
35	SPD_1041 (*nrdH*)	Glutaredoxin-like protein NrdH	Cell redox homeostasis	−150
36	SPD_1046 (*lacG-2*)	6-phospho-beta-galactosidase	Lactose catabolic process via tagatose-6-phosphate	−4.5
37	SPD_1047 (*lacE-2*)	PTS system, lactose-specific IIBC components	Phosphoenolpyruvate-dependent sugar phosphotransferase system	−200
38	SPD_1052 (*lacB*)	Galactose-6-phosphate isomerase subunit LacB	Galactose catabolic process	−2
39	SPD_1050 (*lacD*)	Tagatose 1,6-diphosphate aldolase	lactose catabolic process via tagatose-6-phosphate	−2.1
40	SPD_1051 (*lacC*)	Tagatose-6-phosphate kinase	lactose catabolic process via tagatose-6-phosphate	−1.6
41	SPD_1053 (lacA)	Galactose-6-phosphate isomerase subunit LacA	Galactose catabolic process	−1.7
42	SPD_1083 (*vicX*)	VicX protein	*vicX* may serve as a rho-independent transcriptional terminator	−166
43	SPD_1124 (*licB*)	Protein LicB	Integral component of membrane	−5
44	SPD_1134 (*pyrR*)	Bifunctional protein PyrR	Regulation of transcription	−166
45	SPD_1038 (*phpA*)	Pneumococcal histidine triad protein A	Membrane protein	−200
46	SPD_1292 (*ogt*)	Methylated-DNA–protein-cysteine methyltransferase	DNA dealkylation involved in DNA repair	−200
47	SPD_1339 (*atpF*)	ATP synthase subunit b	ATP synthesis coupled proton transport	−2.0
48	SPD_1341(*atpE*)	ATP synthase subunit c	ATP hydrolysis coupled proton transport	2.26
49	SPD_1357 (*aliB*)	Oligopeptide ABC transporter, oligopeptide-binding protein AliB	Transmembrane transport	−3.78
50	SPD_1373 (*aspC*)	Aminotransferase	Biosynthetic process	−166
51	SPD_1381 (*def-2*)	Peptide deformylase	Translation	−5.55
52	SPD_1626 (*xth*)	Exodeoxyribonuclease III	Endonuclease activity	−200
53	SPD_1642 (*proWX*)	Choline transporter (Glycine betaine transport system permease protein)	Transport	−1.9
54	SPD_1739 (*recA*)	Protein RecA	DNA repair	−5
55	SPD_1757 (*ndk*)	Nucleoside diphosphate kinase	ATP binding	−200
56	SPD_1818 (*comX2*)	Transcriptional regulator ComX1	DNA-templated transcription, initiation	−4.3
57	SPD_1993 (*fucU*)	RbsD/FucU transport protein family protein	Monosaccharide metabolic process	−200
58	SPD_2037 (*cysK*)	Cysteine synthase	Cysteine biosynthetic process from serine	−200
59	SPD_2055 (*guaB*)	Inosine-5′-monophosphate dehydrogenase	GMP biosynthetic process	−4.3
60	SPD_0309 (*luxS*)	S-ribosylhomocysteinase	Quorum sensing (autoinducer-2)	−8.1
61	SPD_1677 (*rafE*)	Sugar ABC transporter, sugar-binding protein	Transport	2.43
62	SPD_0427 (*lacG-1*)	6-phospho-beta-galactosidase	Lactose catabolic process via tagatose-6-phosphate	138.73
63	SPD_0777 (*thiI*)	Probable tRNAsulfurtransferase	Thiamine biosynthetic process	2.60
64	SPD_0874 (*glmU*)	Bifunctional protein GlmU	Cell wall organization	84.33
65	SPD_0877 (*mtnN*)	5′-methylthioadenosine/S-adenosylhomocysteine nucleosidase	Methylthioadenosine nucleosidase activity	171.33
66	SPD_1133 (*pyrB*)	Aspartate carbamoyltransferase	*de novo*' pyrimidine nucleobase biosynthetic process	2.02

The functional annotation of the differentially regulated genes in D39Δ*luxS* and D39 wild-type revealed that the genes involved in DNA replication and repair, ATP synthesis, capsule biosynthesis, cell division and the cell cycle, signal transduction, transcription regulation, competence, virulence, and fructose, lactose and galactose metabolism were down-regulated in the absence of LuxS/AI-2. The fold changes in gene expression in D39Δ*luxS* compared with the D39 wild-type from three independent samples are shown in Table 2.

Galactose metabolism involves the tagatose-6-phosphate and Leloir pathways. Our results revealed that the tagatose-6-phosphate pathway genes (*lacA, lacB, lacC, lacD*, and *lacG-2*), and the Leloir pathway (*galM*) gene were significantly downregulated in D39Δ*luxS*. However, the *lacG-1* gene was upregulated. Furthermore, *lacE2* (encoding the PTS system, lactose-specific IIBC components), *bgaC* (encoding beta-galactosidase 3), and *fucU* (encoding RbsD/FucU transport protein family) were also significantly downregulated. The *cps2A, cps2B, cps2C, cps2D, cps2E, cps2T*, and *cps2G* genes involved in pneumococcal capsule synthesis were significantly downregulated in the D39Δ*luxS*. Similarly, the gene expression levels of the genes encoding DNA replication (*dnaN*), DNA repair (*recJ, recA*, and *ogt*), and ABC transportation (*aliA, aliB, livM*, and *rafE*) were downregulated in the D39Δ*luxS*. The *xth* and *rnr* genes encoding exodeoxyribonuclease III and ribonuclease R were also downregulated. Furthermore, the *luxS* gene mutation downregulated the transcription of regulatory protein-encoding genes such as *ciaR, comX2, blpR, vncR*, and *pyrR*, and ATP synthesis genes (*atpF* and *atpE*). The *ciaR* gene encodes a DNA-binding response regulator protein of the two-component regulatory system known as CiaH/CiaR, which is involved in the early steps of competence regulation (Guenzi et al., 1994), and the *comX2* gene encodes a sigma factor that functions as a competence-specific global transcription modulator involved in bacterial competence.

### Gene expression analysis using real-time RT-PCR

To validate the microarray results, real-time RT-PCR was performed on 15 differentially expressed genes. The 16S rDNA gene was included as a control. The fold changes in gene expression were calculated after normalization of each gene to 16S gene expression levels using the comparative threshold method. The real-time RT-PCR results agree with the microarray shown in Table 3.

**Table 3:** Fold changes in the gene expression of a *Streptococcus pneumoniae luxS* mutant strain (D39Δ*luxS*) during biofilm growth compared with the parental wild-type strain, detected by real-time polymerase chain reaction (PCR).

**Genes**	**Fold change inD39Δ*luxS***
*ply*	1.95
*lytA*	2.2
*ccpA*	0.5
*lacG-2*	0.4
*rnr*	0.64
*argG*	1.25
*comX2*	0.35
*mtnN*	10.35
*bplU*	0.21
*cysK*	0.19
*sufB*	0.24
*aliA*	0.17
*ciaR*	0.27
*glmU*	2.5
*rafE*	6.6

## Discussion

*Streptococcus pneumoniae* asymptomatically colonizes the nasopharyngeal cavity in the form of structures called biofilms (Moscoso and García, 2009). However, bacteria from these biofilms can disperse to other typically sterile sites and causes diseases of the lungs (pneumonia), middle ear (OM), brain (meningitis), and blood (bacteremia) (Hava et al., 2003). The biofilm growth of *S. pneumoniae* is in part regulated by the LuxS/AI QS system (Vidal et al., 2011). Moreover, Trappetti et al. (2011c) reported that LuxS regulates pneumococcal biofilm formation, competence, and fratricide (Trappetti et al., 2011c). However, the effects of *luxS* mutation on global gene expression in pneumococcal biofilms and colonization capability in the middle ear were previously unknown. In the present study, we investigated the effect of *S. pneumoniae luxS* gene mutation on *in vitro* biofilm formation capability and colonization of the rat middle ear mucosa as well as global gene expression in biofilms.

Vidal et al. (2011) detected low *in vitro* biofilm capability in D39Δ*luxS* compared with its parental D39 wild-type strain (Vidal et al., 2011). Herein, we showed that D39Δ*luxS* was unable to form robust early (6–12-h) and late (18–24-h) biofilms. The D39Δ*luxS* biofilms had ~60% less biomass and lower viable bacterial counts. LuxS/AI-2 QS is vital for *in vitro* pneumococcal biofilm growth, and *in vivo* colonization and pathogenesis. In *S. pneumoniae*, AI-2 synthesis from methionine occurs in the activated methyl cycle (AMC), which involves MTA/SAH nucleosidase (Pfs) and LuxS (Parveen and Cornell, 2011). MTA/SAH nucleosidase (Pfs) produces SRH, which is further cleaved by LuxS into homocysteine and 4,5-dihydroxy-2,3-pentanedione, the precursor of AI-2. Herein, we detected increased expression levels of the MTA/SAH nucleosidase-encoding gene, indicating an imbalance due to inactivation of LuxS and high MTA/SAH nucleosidase expression. The products of AMC—such as MTA, SAH, and 5′-deoxyadenosine (5′dADO)—are toxic and product inhibitors that need to be removed for normal growth (Parveen and Cornell, 2011). Therefore, it can be assumed that the low biofilm capability of the *luxS* mutant strain could be caused by the attenuation of AI-2 synthesis and an imbalance in the methionine pathway or AMC due to the accumulation of product inhibitors (Halliday et al., 2010; Vidal et al., 2013).

Another interesting finding was the presence of low levels of EPS in the D39Δ*luxS* biofilms. Although we did not quantify the EPS present in the biofilms, the SEM images revealed that the D39Δ*luxS* biofilms were devoid of EPS, and the bacteria were scattered on the plate.

Pneumococci typically colonize the nasopharyngeal cavities of young children and elderly people. However, under immune-suppressed conditions, these bacteria can disperse to other sterile sites, such as the middle ear, and cause OM. Disease severity depends on the successful colonization of the middle ear mucosa by *S. pneumoniae*. Our *in vivo* results demonstrated that D39Δ*luxS* was less capable of colonizing the rat middle ear mucosa than the wild-type strain. Significantly (> 60%) fewer bacteria were recovered from the rat middle ears inoculated with D39Δ*luxS* compared with the ears inoculated with the D39 wild-type strain. These results indicate that a loss of *luxS* renders bacteria unfit for successful colonization of the rat mucosal membrane. The results also indicate that in *S. pneumoniae*, LuxS plays an important role in the colonization of the rat middle ear, causing OM. Using a pneumonia model, it has been demonstrated that a less virulent *luxS* mutant strain is less likely to spread to the lungs and blood (Stroeher et al., 2003). Previous studies have demonstrated that the LuxS QS system plays an important role in the persistence, virulence, and dissemination of *S. pneumoniae* (Stroeher et al., 2003; Joyce et al., 2004; Vidal et al., 2013). This QS system has been implicated in the persistence of pneumococci in the mouse nasopharynx (Joyce et al., 2004). In pneumococci, LuxS-mediated QS plays an important role in survival and fitness. QS is mediated by a small molecule called auto-inducer-2, which is synthesized by the product of the *luxS* gene in an AMC. The LuxS catalyzes the conversion of SRH to AI-2. Moreover, the LuxS enzyme is absent in humans, and could be an attractive target for novel therapeutic agents against *S. pneumoniae*. Our previous studies showed that blocking the auto-inducer synthesis pathway decreased pneumococcal colonization in the rat middle ear, as well as *in vitro* biofilm formation (Yadav et al., 2012a, 2014). Furthermore, it has been suggested that quorum sensing or auto-inducer activity inhibition may increase the success of antibiotic treatment by increasing the susceptibility of bacterial biofilms and/or by increasing host survival following infection (Wnuk et al., 2009; Brackman et al., 2011).

To further investigate the effect of *luxS* mutation on global gene expression in biofilms, we performed a global gene expression analysis using a cDNA microarray. The expression analysis revealed 117 genes that were differentially expressed in D39Δ*luxS* compared with the D39 wild-type. A large number of genes (60) encoding putative proteins were significantly downregulated, whereas only 6 genes were significantly upregulated. The downregulation of genes encoding DNA replication, repair, cell division, and cell wall protein biosynthesis in the *luxS* mutant indicates that cell division may be perturbed, which results in reduced *in vitro* biofilm formation and *in vivo* colonization capability. Interestingly, a large number of genes encoding ribosomal proteins were downregulated; however, the expression levels were <2-fold (data not shown).

In *S. pneumoniae*, the CSP-mediated QS system plays an important role in biofilm growth, genetic competence, and pathogenesis. The CSP-mediated QS competence system includes the two-component regulatory system CiaH-CiaR, early competence genes (*comAB* and *comCD*), and the late competence gene *comX* (which encodes a global transcription modulator; Ishii et al., 2017). In the present study, we detected a significant reduction in the expression levels of the *ciaR* gene (which encodes the DNA-binding response regulator protein of the two-component regulatory system CiaH/CiaR), and the late competence *comX2* gene. Recently, it has been reported that a *ciaR* gene mutant of *Streptococci sanguinis* was unable to form robust biofilms (Zhu et al., 2017). In *S. pneumoniae*, the ComX alternative sigma factor plays an important role in the initiation of the transcription of the late competence-specific operon, which facilitates DNA uptake and the recombination of DNA (Lee and Morrison, 1999; Luo and Morrison, 2003). Moreover, it has been reported that competence QS plays an important role in biofilm formation, and a *ciaR/H* gene mutant strain is unable to form biofilms (Trappetti et al., 2011b).

The two ABC transporter genes *aliA* and *aliB* were downregulated in the *luxS* mutant (which encodes the Ami-AliA/AliB oligopeptide permease, an ATP-binding cassette transporter that is involved in nutrient uptake). It has been reported that the expression of *aliA* and *aliB* genes is required for nasopharyngeal cavity colonization, and the mutant strain is unable to colonize *in vivo* (Kerr et al., 2004).

The pneumococcal capsule is a major virulence factor, and protects bacteria by interfering with the phagocytic activity of the host (Hyams et al., 2010). The pneumococcal capsule is composed of immunogenic capsular polysaccharides (CPSs) that are encoded by the *cpsABCD* genes (Guidolin et al., 1994; Feldman and Anderson, 2014). In the present study, microarray analysis revealed significantly reduced expression levels of *cps2A, cps2B, cps2C, cps2D, cps2E, cps2T*, and *cps2G* in the D39Δ*luxS* strain compared with the corresponding levels in the D39 wild-type strain. It has been suggested that mutation in each of the *cpsABCD* genes results in a significant reduction in capsule synthesis, as well as reduced virulence and decreased bacterial colonization of the nasopharyngeal cavity (Bender et al., 2003; Morona et al., 2004, 2006). Therefore, blocking LuxS activity probably reduces virulence and renders the bacteria vulnerable to the opsonophagocytic activity of the host (Kim et al., 1999).

The ATP synthesis genes *atpF* and *atpE* were downregulated in the D39Δ*luxS* strain. Membrane-associated F_0_F_1_ H^+^-ATPase is essential for bacteria, and in *S. pneumoniae*, the primary roles of this enzyme are to create a proton gradient using the energy provided by ATP hydrolysis, and to maintain intracellular pH via proton extrusion (Martín-Galiano et al., 2001). In *S. pneumoniae*, the activity of the F_0_F_1_ ATPase increases as the pH of the growth medium decreases. Regulation of this pH-inducible phenotype occurs at the level of transcription initiation (Martín-Galiano et al., 2001). In streptococci, the F_0_F_1_ H^+^-ATPase-encoding operon is *atpEBFHAGDC*, and the F0 gene order is *atpEBF* (Shabayek and Spellerberg, 2017).

**Figure 8:**
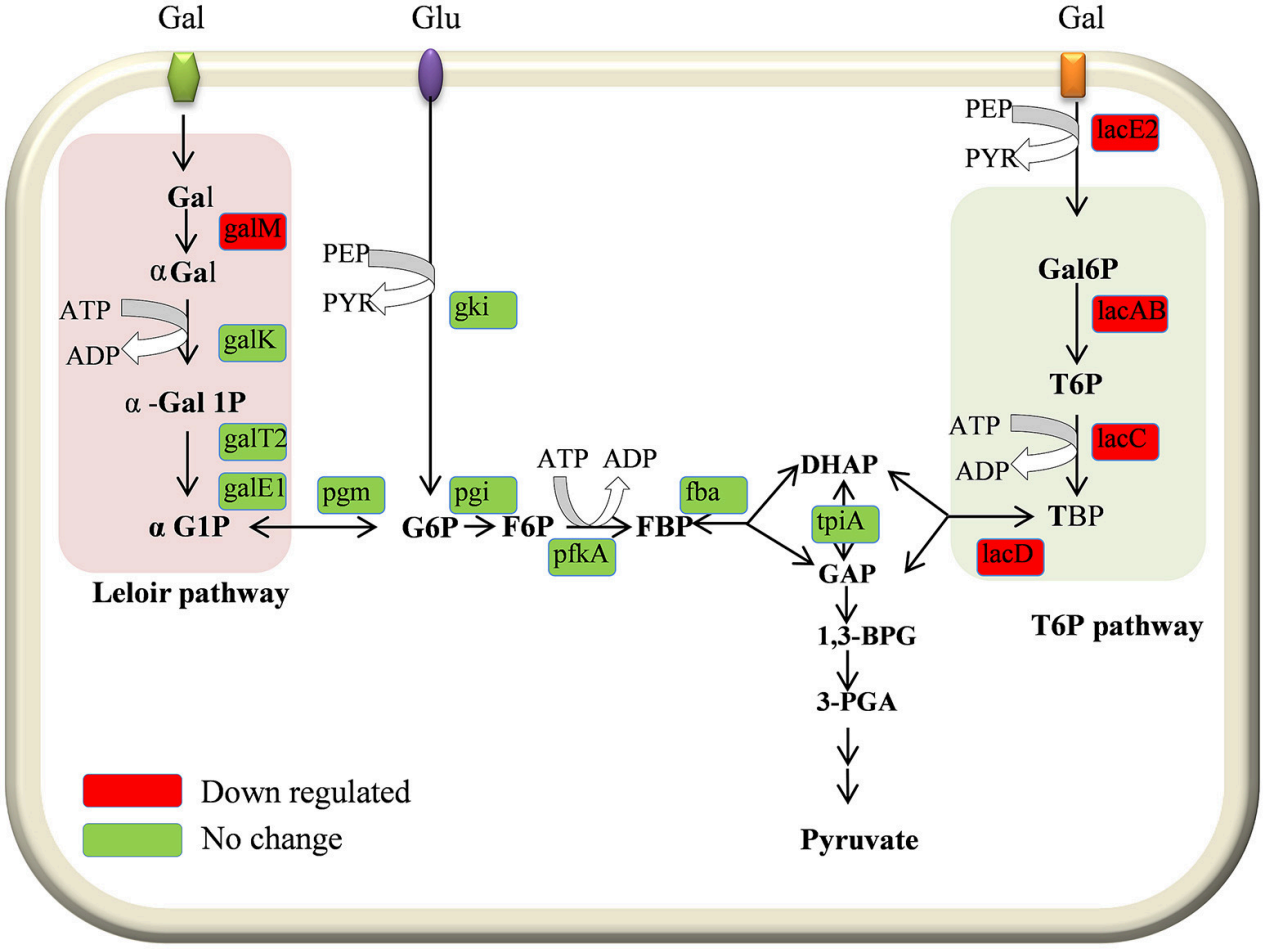
Schematic representation of galactose metabolism pathway in *Streptococcus pneumoniae* D39. In *S. pneumoniae*, lactose, and galactose are metabolized by the tagatose-6-phosphate pathway (light gray box) and the Leloir pathway (left; light pink box). The *lacA, lacB, lacC*, and *lacD* genes are involved in the tagatose-6-phosphate pathway, and the *galM, galK, galT-1*, and *galE-2* genes are involved in the Leloir pathway. The *lacE2* gene (which encodes the PTS system, lactose-specific IIBC component) is required for galactose transport. The *lacA, lacB, lacC, lacD, galM*, and *lacE-2* genes were downregulated in the present study.

In the present study our results demonstrated the downregulation of galactose pathway genes, such as *lacB, lacC, lacD*, and *lacA* (of the T6P pathway), and *galM* (of the Leloir pathway) in the D39Δ*luxS* strain (Figure 8). In addition, *lacE2* (encoding the PTS system, lactose-specific IIBC components), *bgaC* (encoding beta-galactosidase 3), and *fucU* (encoding the RbsD/FucU transport protein family) were also downregulation in D39Δ*luxS*. Pneumococci can utilize various carbohydrate sources during colonization of the nasopharyngeal cavity (Buckwalter and King, 2012; Yadav et al., 2013). It has been reported that galactose catabolic route genes such as *lacAB* and *lacD* (of the T6P pathway) and *galM* (of the Leloir pathway) are required for pneumococcal colonization, and were upregulated during bacteria growth on mucin (Paixão et al., 2015). Furthermore, it has been suggested that *lacD* (T-6-p) or the Leloir pathway mutant D39 are less capable of colonizing the murine nasopharynx and have reduced virulence (Paixão et al., 2015). Recently, Trappetti et al. (2017) suggested that LuxS/AI-2 signaling enables pneumococci to utilize galactose as a carbon source, and enhances capsular polysaccharide production and the hyper-virulent phenotype (Trappetti et al., 2017). Present and previous study results demonstrate that the loss of functional LuxS hinders carbohydrate utilization, leading to the reduced colonization capability of pneumococci *in vivo*.

## Conclusion

The results of this study demonstrate that the *S. pneumoniae* D39Δ*luxS* strain has a reduced ability to form early and late *in vitro* biofilm, and is less capable of colonizing the rat middle ear mucosa. LuxS/AI-2 regulates the expression of the genes involved in cell division and growth, capsule synthesis, carbohydrate metabolism, competence, virulence, and bacterial fitness during colonization.

## Author contributions

MY, J-JS, S-WC, YG, SK and JV conceived and designed the experiments. MY and SK performed the experiments. MY, J-JS, S-WC, YG, and JV analyzed the data; S-WC, YG, J-JS, SK, and JV contributed reagents, materials, analysis tools. MY, J-JS, and JV wrote the paper.

### Conflict of interest statement

The authors declare that the research was conducted in the absence of any commercial or financial relationships that could be construed as a potential conflict of interest.
